# Causality between sarcopenia and diabetic neuropathy

**DOI:** 10.3389/fendo.2024.1428835

**Published:** 2024-09-13

**Authors:** Yi Fang, Xiaoqing Yuan, Qing Zhang, Juan Liu, Qing Yao, Xinhua Ye

**Affiliations:** ^1^ Department of Endocrinology, The Affiliated Changzhou No. 2 People’s Hospital of Nanjing Medical University, Changzhou, China; ^2^ Changzhou Medical Center, The Affiliated Changzhou No. 2 People’s Hospital of Nanjing Medical University, Changzhou, China

**Keywords:** diabetic neuropathy (DN), diabetes, sarcopenia, causality, Mendelian randomization (MR), cross-sectional study, correlation

## Abstract

**Background:**

Past studies have demonstrated that diabetic neuropathy is related to sarcopenia, but the further causal relation is still unclear. We sought to investigate the causal relationship by combining data from cross-sectional and Mendelian randomization (MR) studies.

**Methods:**

The genome-wide association studies data were collected from the UK Biobank and the European Working Group on Sarcopenia to conduct a bi-directional two-sample MR study to explore the causality between diabetic neuropathy and relevant clinical traits of sarcopenia, including appendicular lean mass (ALM), walking speed and low hand grip strength. The inverse-variance weighted and various sensitivity analyses were used to obtain MR estimates. We also enrolled a total of 196 Type 2 diabetes patients from April 2021 to April 2024 and divided them into the Distal peripheral neuropathy (DPN) group (n=51) and non-DPN group (n=145) via vibration perception threshold (VPT) and neuropathy deficit score. Logistic regression and ROC curve analysis were used to investigate the relationship between DPN and relevant sarcopenia clinical features.

**Results:**

According to a forward MR analysis, decreased walking speed (OR: 0.04, 95% confidence interval (CI): 0.01-0.16; P<0.001) and increased ALM (1.25 [1.05-1.50], P=0.012) had a causal effect on developing diabetic neuropathy. According to reverse MR results, developing diabetic neuropathy had a causal effect on decreased walking speed (0.99 [0.99-1.00], P=0.007) and low grip strength (1.05 [1.02-1.08], P<0.001). The cross-sectional study showed that 5-time stand time (P=0.002) and 6-meter walking speed (P=0.009) had an inverse association with DPN. Additionally, we discovered that ASMI (P=0.030) and 5-time stand time (P=0.013) were separate risk factors for DPN.

Conclusion

The MR study suggested that diabetic neuropathy may have a causality with relevant clinical traits of sarcopenia, and our cross-sectional study further proved that sarcopenia indexes are predictors of diabetic neuropathy.

## Introduction

Type 2 diabetes (T2DM) is a chronic disease established primarily by disturbances in glucose metabolism, accompanied by metabolic disorders in fats, proteins, and other aspects (1). Diabetic neuropathy is a prevalent form of neuropathy, affecting approximately half of all diabetes patients, resulting in significant mortality rates and imposing substantial economic burdens. diabetic neuropathy is a common chronic complication of T2DM, that primarily affects the peripheral nervous system, leading to disruptions in in motor, sensory, and autonomic nerve functions (2). Distal peripheral neuropathy (DPN) is a type of diabetic neuropathy affecting the peripheral nerves, primarily manifesting as tingling, numbness, and weakness in the hands and feet due to diabetes. It can result in foot infections, foot ulcers, gangrene, and even amputation, significantly impacting the patient’s quality of life and serving as a major cause of disability and death (3, 4). Nerve conduction tests serve as the current gold standard for diagnosing DPN. Nonetheless, they are lengthy, costly, and challenging to incorporate into regular clinical practice. So clinical screening for diabetic neuropathy relies mainly on five examinations, including vibration perception, pinprick sensation, ankle reflex, pressure sensation, and temperature sensation (5, 6).

The vibrating perception threshold (VPT) test, due to its non-invasiveness, convenience, economy, and quantitative advantages, has become a crucial tool for diagnosing and assessing DPN, both domestically and internationally (7–10). The sensitivity of VPT in diagnosing DPN ranges from 77.3% to 100.0%, with a specificity of 72.8% to 81.0%, and it has been widely applied in clinical research related to DPN (11–13).

Sarcopenia refers to the phenomenon where muscle function and mass gradually decrease with increasing age (14). Sarcopenia is closely associated with mobility impairment, falls, low bone density, and metabolic disorders, making it a significant cause and manifestation of physiological decline in older adults. It can impact their quality of life and increase hospitalization rates and medical expenses for them (15). A large community study in the Czech Republic revealed that direct medical costs for older adults with sarcopenia were more than twice as high as those without sarcopenia (16). Another study demonstrated that sarcopenia is associated with increased hospital costs (17).

Recently, the relationship between sarcopenia and diabetic neuropathy has garnered significant attention. Studies have increasingly highlighted a strong association between these two conditions. Henning et al. reported that individuals with diabetes exhibit reduced lower limb muscle volume, which may be linked to the severity of neuropathy (18). Christer et al. suggested that impaired nerve function can lead to denervation and a consequent decrease in muscle strength (19). Similarly, Giorgio et al. indicated that DPN affects muscles by impairing motor nerve conduction (20). Cloin et al., revealed a significant reduction in both muscle mass and contractile strength in patients with DPN (21). Yasemin et al., indicated that Sarcopenia increases the risk of developing diabetic neuropathy (22). While another study proposed that diabetic neuropathy might be a potential risk factor for sarcopenia (23). Furthermore, a systematic review and meta-analysis confirmed a significant association between DPN and sarcopenia (24). Although previous studies have shown a significant association between diabetic neuropathy and sarcopenia, no research has definitively proven a causality between them.

Mendelian randomization (MR) utilizes genetic variants, typically single-nucleotide polymorphisms (SNPs), as instrumental variables to determine the causality between lifelong exposure and disease outcomes. This method is highly reliable because it is less prone to reverse causality and residual confounding factors. Unlike the associations derived from traditional cross-sectional studies, the causality drawn from MR analyses are considered more reliable, especially when their results align with those of conventional cross-sectional studies (25, 26). Therefore, we carried out a MR study to resolve this dispute and conducted a cross-sectional study between DPN and sarcopenia to further provide the relationship between them. This research may offer a theoretical foundation for preventing these two conditions.

## Materials and methods

### Mendelian randomization analysis design

**Figure 1:**
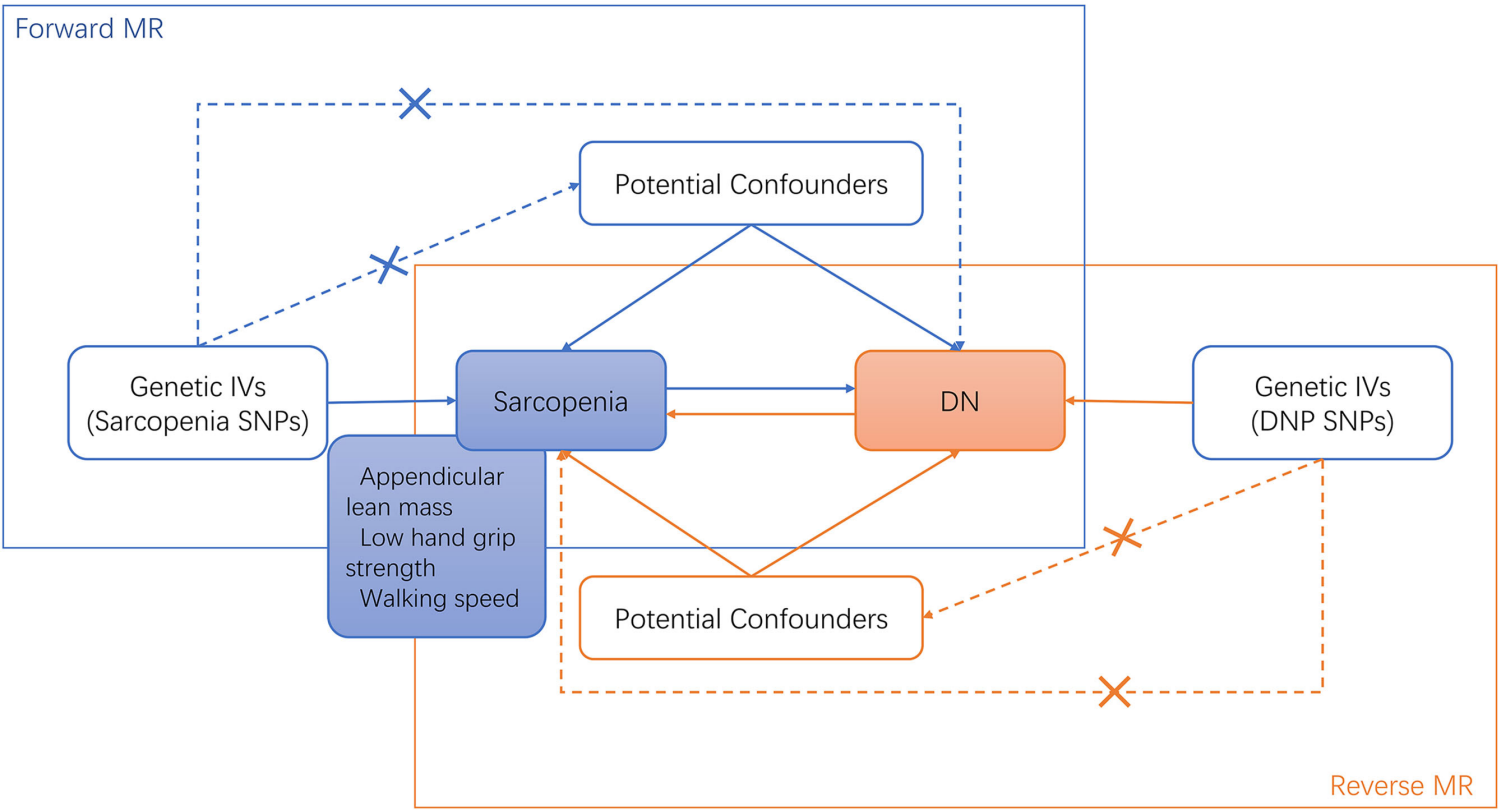
Bi-directional MR study analysis design of sarcopenia and diabetic neuropathy. IVs, instrumental variants; SNP, single nucleotide polymorphism; DN, diabetic neuropathy.

Based on the STROBE-MR guidelines, we used genetic variants as IVs for the MR analysis in this current study (25). The MR analysis utilizes instrumental variables (IVs) based on genetic variation. The three fundamental assumptions of MR studies are (1): the relevance assumption, which posits that genetic variants are associated with the risk factor (2); the independence assumption, which asserts that the genetic variant is independent of confounders; and (3) the exclusion restriction, meaning the genetic variant affects the outcome solely through the risk factor. The specific details of each method are described in detail by Liu et al (27). The framework of the current MR study was described in **Figure 1**.

### Data sources description

Aggregate grip strength data collected from genome-wide association Studies (GWAS) came from a meta-analysis involving 22 cohorts of 256,523 Europeans aged 60 years or older (28). Sarcopenia was identified in 48,596 participants (18.9%) based on EWGSOP criteria (grip strength <30 kg for men and <20 kg for women). Appendicular lean mass (ALM) data for 450,243 patients and walking speed data for 459,915 patients were collected from the UK Biobank’s GWAS database and pooled for analysis (29). Additionally, GWAS summary data for diabetic neuropathy were obtained from the IEU Open GWAS project, including 1,415 European cases and 162,201 European controls. The details of the GWAS data included in this study were shown in **Supplementary Material 1**.

### Selection of genetic instrumental variables

To screen for SNPs that fit the MR Hypothesis, a series of approaches were taken. First, SNPs associated with sarcopenia-related clinical traits need to reach a threshold of P < 5 × 10^-8^ to achieve genome-wide significance. To enlarge SNPs, the threshold of diabetic neuropathy-related SNPs was expanded to P < 5 × 10^−6^ according to research by Liu et al (27). Then, SNPs in strong LD were excluded by using the linkage disequilibrium (LD) clustering algorithm with r^2^ < 0.001, aggregation distance = 10,000 kb, and value of P < 5 × 10^−8^ (30). Furthermore, the F statistic was used to test the strength of the allele scores as instruments, the calculation formula is as follows:


$$\text{F=}\frac{N-K-1}{\text{K}}\times \frac{{\text{R}}^{2}}{{1-R}^{2}}$$

N is the sample size of the exposed dataset, K is the number of SNPs, R^2^ is the proportion of the variation explained by SNPs, and F < 10 was excluded (31). After removing SNPs, the MR Pleiotropy RESidual Sum and Outlier (MR-PRESSO)

method (3000 repeated settings) was used to find potential outliers (32). Subsequently, palindromic SNPs were removed by combining the exposed datasets with DN datasets. Through these rigorous screening, the remaining SNPs were used for subsequent analysis. Detailed information on these SNPs can be found in **Supplementary Materials 2****–****7**.

### MR statistical analysis and data visualization

The bias of the weak instrumental variables was evaluated by calculating the F-statistic. The formula for calculating F-statistic was consistent with Burgess et al (31),. F > 10 was considered indicative of a strong instrumental variable (31). We used the MR-PRESSO method (3000 repeated settings) to find outliers (32). Then outliers were eliminated so that the data could be re-evaluated.

In the MR analysis, the inverse variance weighted (IVW) approach is known for its efficiency and minimal bias in estimating causal effects, provided that the underlying assumptions are met (33). Additionally, the weighted median method was utilized in this analysis. The weighted median method calculates the causal effect by taking the median of the estimates from individual instrumental variables, weighted by the inverse of their variance (34). This robust approach provides reliable causal estimates even if up to 50% of the genetic instruments are invalid or influenced by pleiotropy (35, 36). To supplement the analysis, the MR egger, MR egger (bootstrap) and weighted mode methods were also applied as additional reference standards.

We performed a series of sensitivity analyses, including Cochran’s Q test, MR Egger intercept test, and leave-one-out analysis, to measure the dependability of the results. Heterogeneity was also determined using Cochran’s Q test. Pleiotropy was evaluated using the MR-Egger intercept test and the Leave-one-out analysis. The “TwoSampleMR” and “MR-PRESSO” packages of the R software (version 4.3.1) were used to implement all MR analyses as well as sensitivity analyses.

### Clinical study data

**Figure 2:**
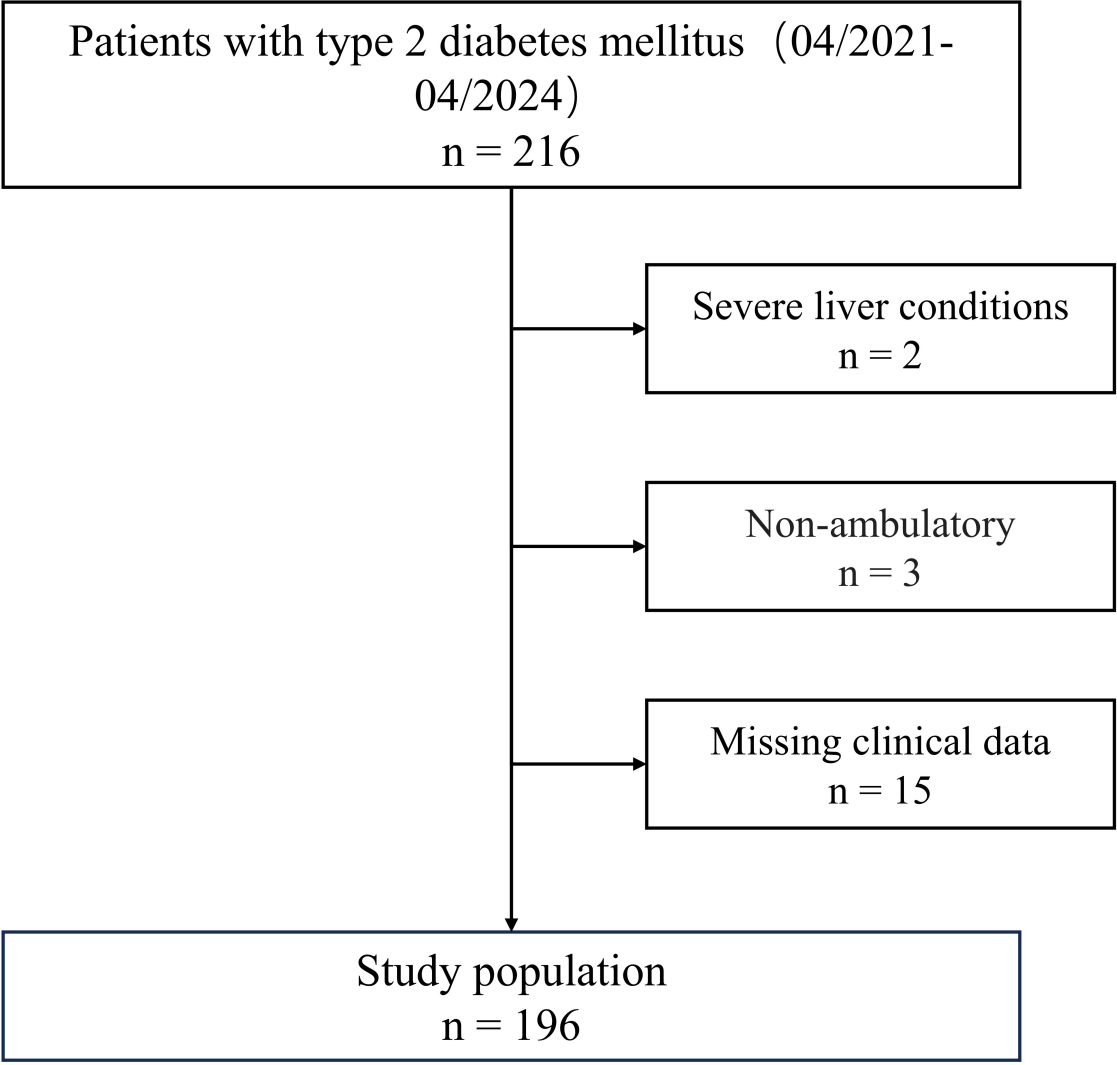
Flowchart of Participant Inclusion and exclusion.

A total of 196 hospitalized patients with T2DM, aged 60 to 80 years, who underwent VPT examinations at our department from April 2021 to April 2024 were included in this study. Then we then excluded patients that met any of the following criteria (1): severe liver conditions (liver enzyme ALT≥3-fold the upper limit of normal range) (n = 2) (2); severe renal dysfunction (estimated glomerular filtration rate [eGFR]<30 mL/min/1.73m^2^) (3); respiratory insufficiency (PaO_2_< 60 mmHg or PaCO_2_> 45 mmHg) (4); sepsis (5); cognitive impairment (6); non-ambulatory (n = 3); and (7) missing clinical data (n = 15). The flowchart of the study population is presented in **Figure 2**.

All patients met the diagnostic criteria for T2DM according to the WHO 2019 diabetes guidelines. DPN was diagnosed by the American Diabetes Association’s 2005 diagnostic criteria if the vibration perception threshold VPT was over 25 volts accompanied by a positive Neuropathy Deficit Score (37). Based on these criteria, T2DM patients are categorized into DPN (n=51) and non-DPN (n=145) groups based on the results of VPT tests and clinical signs.

### Medical history and anthropometric and biochemical analyses

General information was collected including the patient’s age, sex, duration of diabetes, smoking, alcohol consumption, and co-occurrence of hypertension, etc. The anthropometric analysis included weight, height, and bio-impedance analysis (Inbody Co., Ltd. Korea), and the Body mass index (BMI) was calculated accordingly. Fasting blood and morning urine samples were collected following a more than 8-hour overnight fast. Fasting plasma glucose (FPG), fasting insulin (FINS), glycated hemoglobin (HbA1c), total cholesterol (TC), triglyceride (TG), serum high-density lipoprotein (HDL-c), serum low-density lipoprotein (LDL-c), creatinine and blood urea nitrogen (BUN) were assessed by the protocol of the central laboratory.


HOMA-IR=(FINS×FPG)/22.5

### Neuropathy evaluation

The same technician assessed VPT using Horwell Neurothesiometers (Wilford Industrial, Nottingham). The mean VPT values of both lower limbs were selected for our analysis.

### Sarcopenia assessment

Technicians assessed hand grip strength with a computerized grip strength dynamometer (TSINGHUA TONGFANG, China) after requesting subjects to refrain from strenuous exercise before the measurement (38). Measurements were obtained repeatedly for every hand, and the dominant hand’s mean value was utilized for analysis. The 2019 Asian Working Group defined the relevant clinical traits of sarcopenia for Sarcopenia criteria (39). The parameter of muscle mass index was the appendicular skeletal muscle mass (ASM) divided by the height squared (m^2^) (ASMI). Low muscle strength and muscle mass were defined as values below the cutoffs of 28 kg and 7.0 kg/m2, respectively, for men and 18 kg and 5.7 kg/m2, respectively, for women. Criteria for low physical performance are 6-m walk <1.0 m/s or 5-time chair stand test ≥12 seconds.

### Clinical study statistical analysis

Statistical analysis was performed using SPSS 25.0. Different tests were employed depending on the type of data: independent samples t-test for normally distributed measurements, Kruskal–Wallis H-test for non-normally distributed measurements, chi-square test for count data, and logistic regression model for analyzing the risk factors for DPN. Simple correlations between VPT and various factors were evaluated by Pearson or Spearman correlation analysis. All statistical tests were tailed twice, and p<0.05 was considered statistically significant.

## Results

### The forward MR analysis showed that parts of the relevant clinical traits of sarcopenia had causal effects on diabetic neuropathy

The forward MR analysis showed that decreased walking speed had a causal effect on developing diabetic neuropathy via IVW (OR: 0.04, 95% confidence interval (CI): 0.01-0.16; P<0.001) and weighted median analysis (0.07 [0.01-0.48], P = 0.006). And increased ALM was proved to have a causal effect on diabetic neuropathy by IVW (1.25 [1.05-1.50], P = 0.012), weighted median (1.37 [1.04-1.80], P = 0.026), weighted mode (2.08 [1.08-4.03], P = 0.030) and MR egger (bootstrap) analysis (1.47 [1.04-2.10], P = 0.017). However, low grip strength did not exhibit any direct influence on the development of diabetic neuropathy across all MR analysis methods.

**Figure 3:**
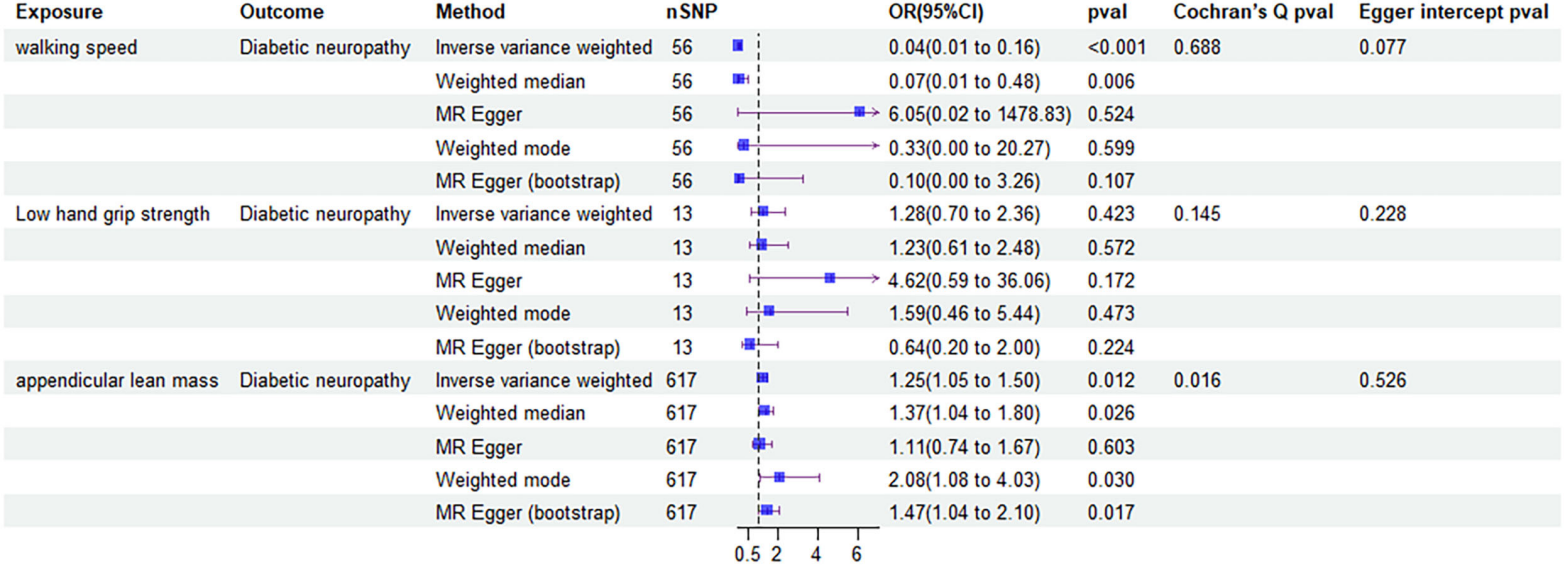
Forest plot of MR results of the effect of sarcopenia related clinical traits on diabetic neuropathy.

**Figure 4:**
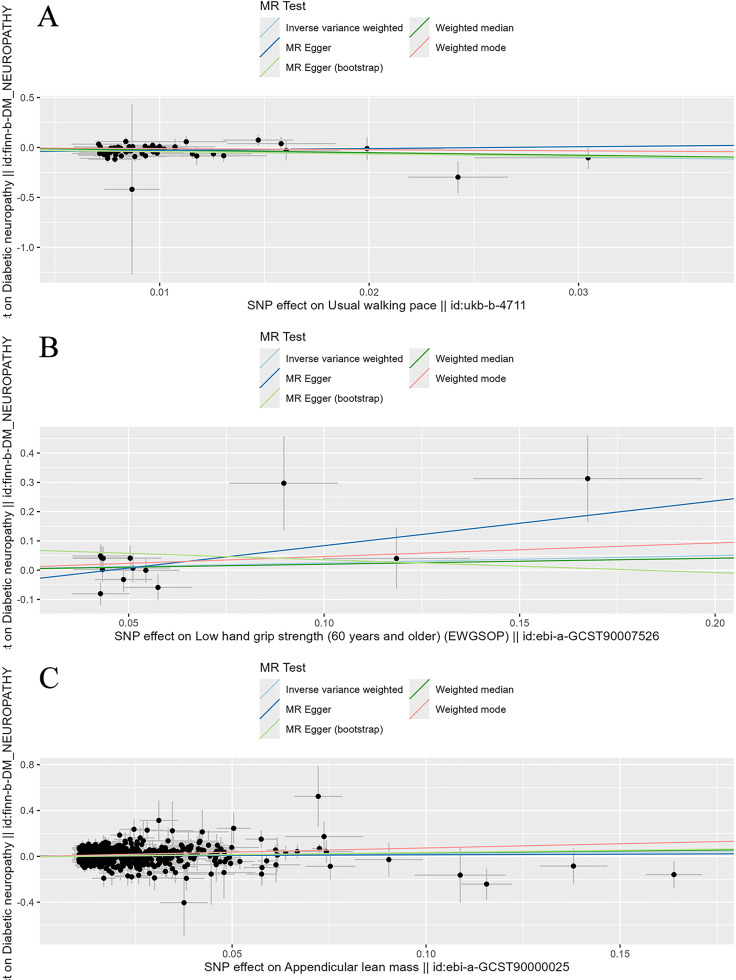
Scatter plot of the causality of sarcopenia related clinical traits on diabetic neuropathy. **(A)** The causality of walking speed on diabetic neuropathy. **(B)** The causality of low hand grip strength on diabetic neuropathy. **(C)** The causality of ALM on diabetic neuropathy.

To evaluate the stability and reliability of each MR method, we employed Cochran’s Q test, MR-Egger intercept test, and leave-one-out analysis. Cochran’s Q test identified heterogeneity in ALM (P < 0.05), suggesting that SNPs from different database or population may influence the result. However, it was considered acceptable due to the application of random-effects IVW as the primary method. All Egger intercept p-values > 0.05 indicated no presence of horizontal pleiotropy as per MR-Egger intercept tests. The forest plot (**Figure 3**) and scatter plot (**Figure 4**) offer a detailed depiction of all the above results.

**Figure 5:**
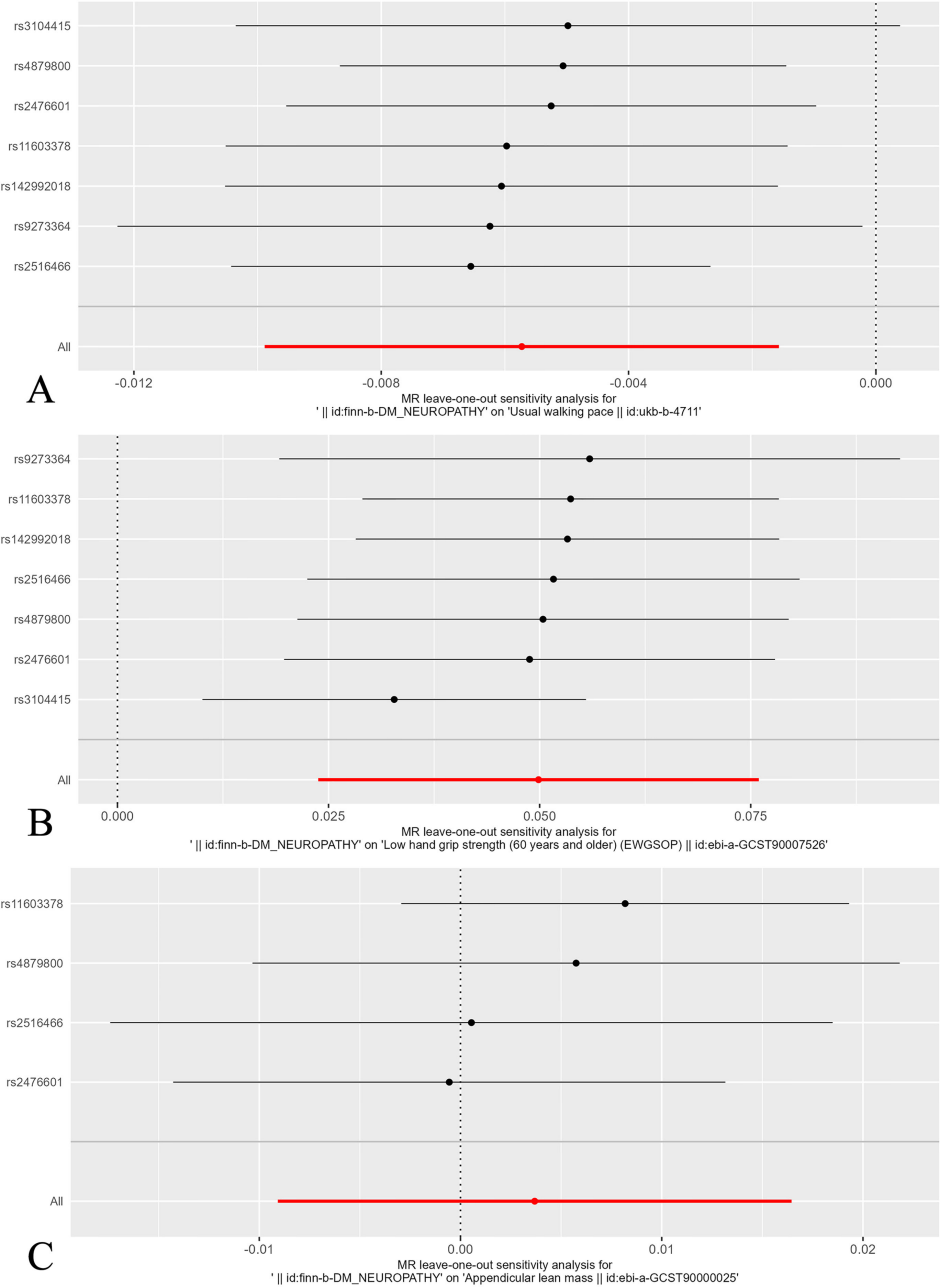
Leave-one-out plot of the effect of sarcopenia related clinical traits on diabetic neuropathy. **(A)** Leave-one-out plot of the effect of walking speed on diabetic neuropathy. **(B)** Leave-one-out plot of the effect of low hand grip strength on diabetic neuropathy. **(C)** Leave-one-out plot of the effect of ALM on diabetic neuropathy.

Leave-one-out analysis confirmed that no SNPs significantly affected the results. **Figure 5** shows the leave-one-out analysis results detailly.

### The reverse MR analysis revealed that diabetic neuropathy had a causal effect on the certain clinical traits of sarcopenia

The four MR analysis methods that demonstrated a causal relationship between developing diabetic neuropathy and walking speed included IVW (0.99 [0.99-1.00], P = 0.007), weighted median (0.99 [0.99-1.00], P = 0.001), weighted mode (0.99 [0.99-1.00], P = 0.013) and MR egger (bootstrap) analysis (0.99 [0.99-1.00], P = 0.021). All MR analysis methods confirmed that diabetic neuropathy had causal effects on low hand grip strength, but no causal effects on ALM.

**Figure 6:**
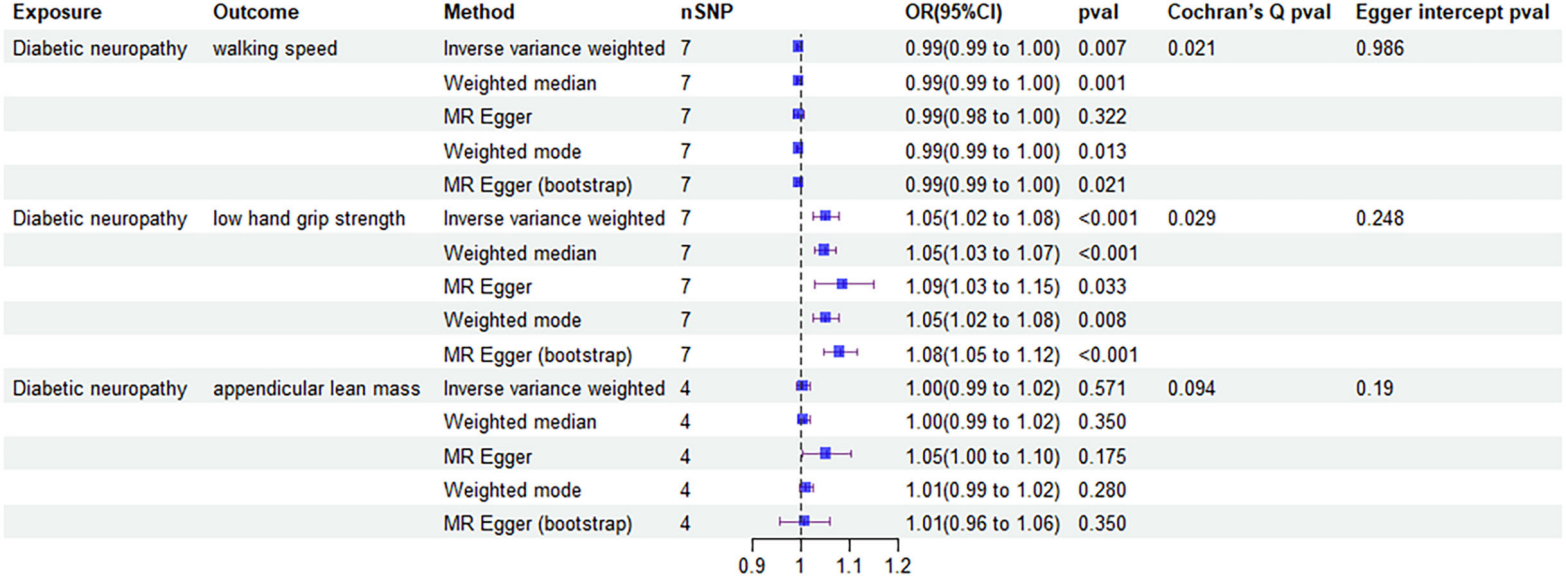
Forest plot of MR results of the effect of diabetic neuropathy on sarcopenia related clinical traits.

**Figure 7:**
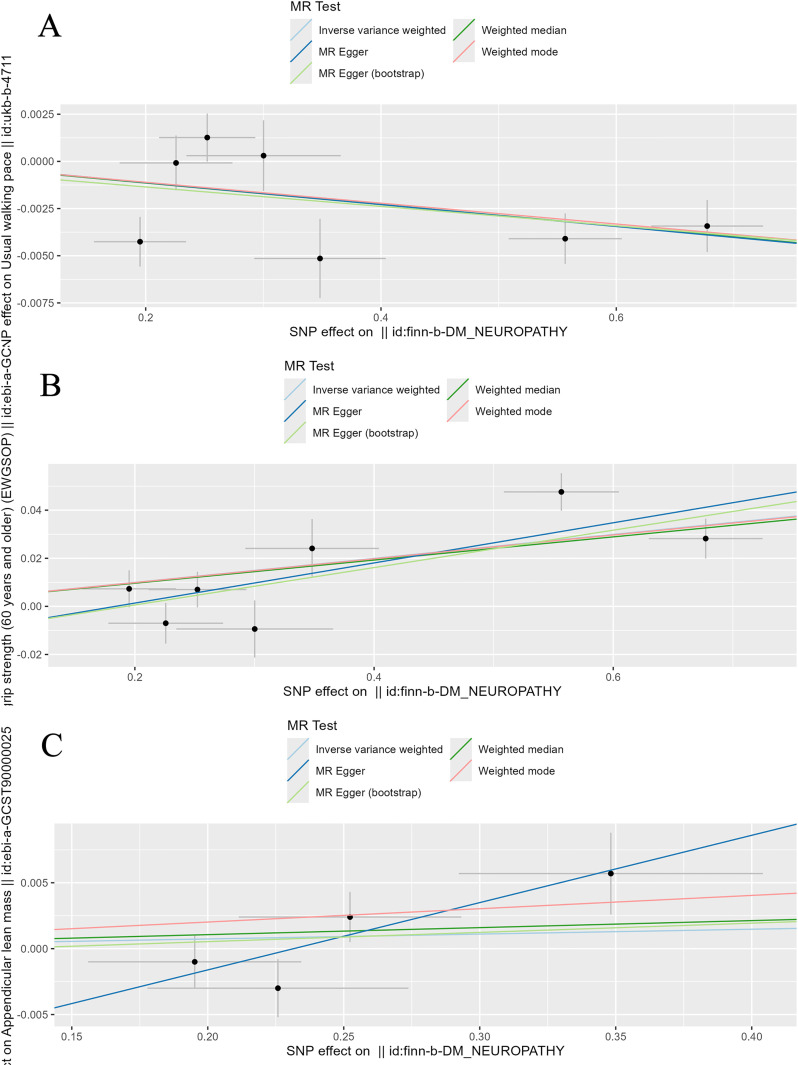
Scatter plot of the causality of diabetic neuropathy on sarcopenia related clinical traits. **(A)** The causality of diabetic neuropathy on walking speed. **(B)** The causality of diabetic neuropathy on low hand grip strength. **(C)** The causality of diabetic neuropathy on ALM.

**Figure 8:**
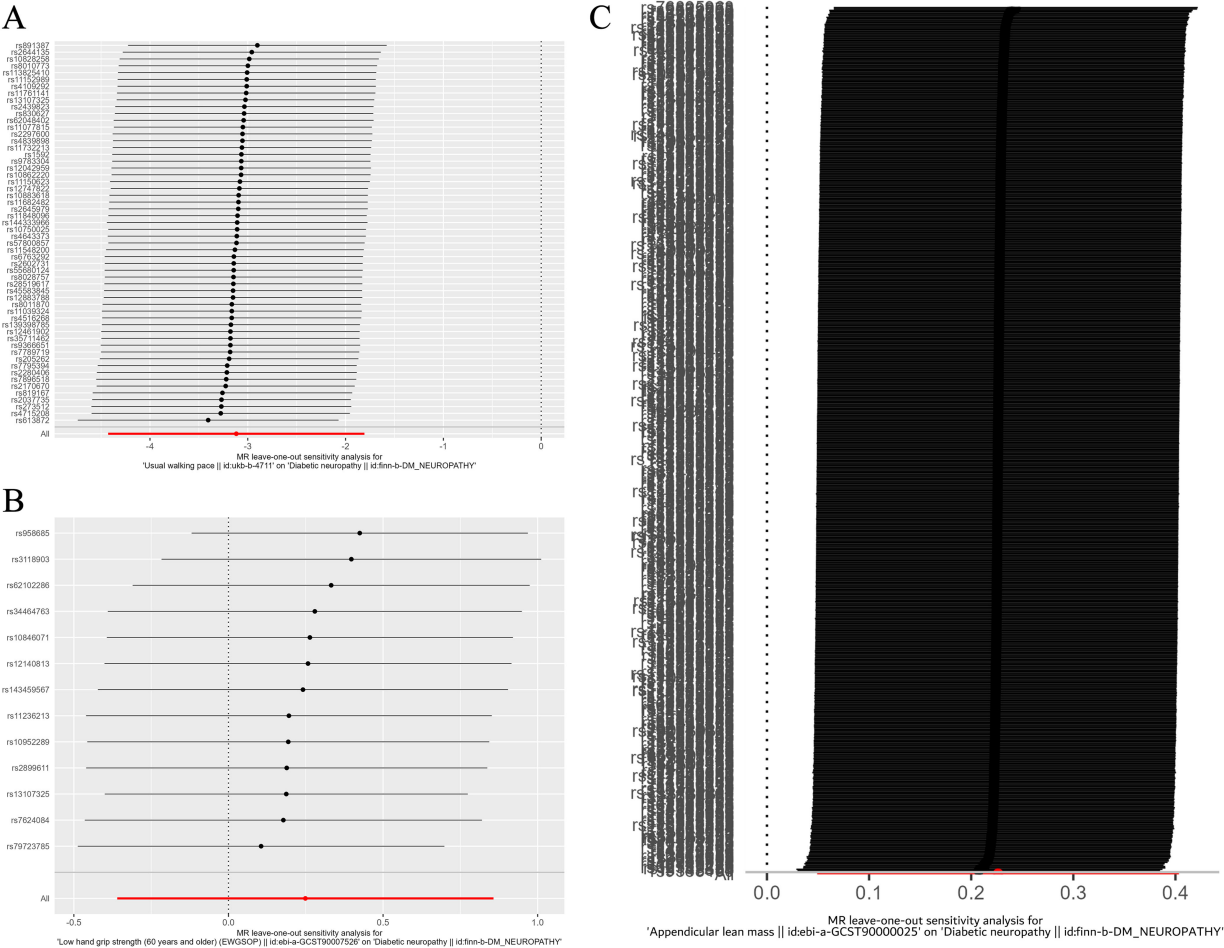
Leave-one-out plot of the effect of diabetic neuropathy on sarcopenia related clinical traits. **(A)** Leave-one-out plot of the effect of diabetic neuropathy on walking speed. **(B)** Leave-one-out plot of the effect of diabetic neuropathy on low hand grip strength. **(C)** Leave-one-out plot of the effect of diabetic neuropathy on ALM.

Then, we assessed the results in the same manner as the forward MR analysis above. Cochran’s Q test indicated that there was heterogeneity in ALM, but it was also considered acceptable due to the application of random-effects IVW as the primary method. (**Figures 6**, **7**). MR-Egger intercept tests and leave-one-out analysis produced results consistent with the forward MR analysis above (**Figure 8**).

### Baseline patient features

A total of 196 patients aged ≥60 years with T2DM were enrolled in this study, who were then divided into DPN (n=51) and non-DPN (n=145) groups. The DPN prevalence was determined as 26% (51/196). As it was seen in **Table 1**. Significant variations were identified between patients with and without DPN in terms of sex, age, weight, alcohol consumption, hypertension comorbidity, as well as levels of HbA1c, fasting insulin, HDL-c, creatinine, and VPT (P<0.001). Compared to the patients without DPN, the patients who had DPN were older, more obese, and with higher HbA1c, creatinine, and lower fasting insulin. Moreover, the number of males, alcohol consumers, and individuals with hypertension was more in patients with DPN than the ones without DPN (P<0.05). Additionally, in terms of muscle-related indicators, the patients with DPN had longer 5-time chair stand time and slower 6-meter walking speed, while their grip strength and ASMI showed no significant difference.

**Table 1:** Baseline characteristics of patients with T2DM with or without diabetic peripheral neuropathy.

	DM	DM-DPN	t/z/c²	p
**Patients (n, %)**	145,74.0	51,26.0	–	–
**Male (n, %)**	64,44.1	33,64.7	6.385	0.012
**Age (y)**	67.00(62.50,70.00)	71.00(66.00,74.00)	3.768	<0.001
**Body weight (kg)**	62.32 ± 9.95	66.02 ± 10.99	2.220	0.028
**BMI (kg/m2)**	23.84 ± 3.11	24.02 ± 2.83	0.361	0.719
**Diabetes duration (y)**	12.65 ± 7.61	13.65 ± 9.21	0.758	0.449
**Smokers (n, %)**	27,18.6	16,31.4	3.582	0.058
**Drinker (n, %)**	14,9.7	12,23.5	6.312	0.012
**Combined hypertension (n, %)**	88,60.7	41,80.4	6.510	0.011
**FPG (mmol/l)**	7.24 ± 2.24	7.46 ± 2.43	0.597	0.551
**HbA1c (%)**	8.69 ± 1.80	9.51 ± 2.00	2.702	0.008
**FINS (μU/mL)**	33.61(19.59,54.08)	23.02(15.47,39.42)	1.966	0.049
**HOMA-IR**	1.49(0.80,2.46)	1.15(0.69,2.09)	1.252	0.210
**Triglyceride (mmol/L)**	1.43(1.03,2.14)	1.55(1.10,2.23)	0.690	0.490
**Total Cholesterol (mmol/L)**	4.28 ± 1.01	4.10 ± 1.12	1.029	0.305
**HDL-c (mmol/L)**	1.13 ± 0.28	1.03 ± 0.26	2.222	0.027
**LDL-c (mmol/L)**	2.41 ± 0.74	2.32 ± 0.85	0.731	0.466
**Creatinine (mmol/L)**	60.75(52.00,73.98)	69.60(55.90,87.00)	2.498	0.013
**BUN (mmol/L)**	6.40(5.30,7.50)	6.50(5.50,8.30)	1.100	0.271
**VPT (V)**	16.03 ± 4.59	28.48 ± 2.16	25.556	<0.001
**Grip strength (kgf)**	25.60(20.10,34.65)	26.80(21.60,35.10)	0.762	0.446
**5-time chair stand test (s)**	10.11(8.71,11.65)	11.61(9.20,14.22)	3.105	0.002
**6-m walk speed (m/s)**	1.17 ± 0.27	1.06 ± 0.26	2.624	0.009
**ASMI (kg/m2)**	6.89 ± 1.29	7.23 ± 1.13	1.649	0.101

Data were shown as mean ± SD, median (interquartile range), or n (%) as appropriate.

BMI, body mass index; FPG, fasting plasma glucose; HbA1c, glycosylated hemoglobin; FINS, fasting insulin; HDL-c, serum high-density lipoprotein; LDL-c, serum low-density lipoprotein; BUN, blood urea nitrogen; VPT, vibrating perception threshold; ASMI, appendicular skeletal muscle mass index.

### Associations of sarcopenia indexes with DPN

The logistic regression analysis results indicated that sex (5.840 [1.444-23.618], P = 0.013), age (1.146 [1.047-1.254], P = 0.003), body weight (1.137 [1.020-1.267], P = 0.021), alcohol drinking (3.146 [1.026-9.648], P = 0.045), HbA1c (2.211 [0.851-5.748], P = 0.045), 5-time chair stand test (1.230 [1.045-1.448], P = 0.013), and ASMI (0.337 [0.126-0.902], P = 0.030) were all independent risk factors for DPN in patients with T2DM (**Table 2**).

**Table 2:** Logistic regression analysis of diabetic peripheral neuropathy risk factors.

	OR	95%CI	p
**Gender**	5.840	1.444-23.618	0.013
**Age**	1.146	1.047-1.254	0.003
**Body weight**	1.137	1.020-1.267	0.021
**Drinker**	3.146	1.026-9.648	0.045
**Combined hypertension**	2.211	0.851-5.748	0.103
**HbA1c**	1.321	1.062-1.643	0.012
**FINS**	1.003	0.993-1.014	0.546
**HDL-c**	0.591	0.129-2.700	0.497
**Creatinine**	1.004	0.987-1.022	0.636
**Grip strength**	0.986	0.924-1.053	0.677
**5-time chair stand test**	1.230	1.045-1.448	0.013
**6-m walk speed**	1.742	0.219-13.851	0.600
**ASMI**	0.337	0.126-0.902	0.030

OR, odds ratio; 95% CI, 95% confidential interval; HbA1c, glycosylated hemoglobin; FINS, fasting insulin; HDL-c, serum high-density lipoprotein; ASMI, appendicular skeletal muscle mass index.

### Risk factors for diabetic neuropathy

The correlation analysis between VPT and various factors in T2DM patients showed that VPT was positively related to sex (r: 0.164, P = 0.022), age (r: 0.363; P<0.001), body weight (r: 0.181, P = 0.011), Combined hypertension (r: 0.166, P = 0.020), HbA1c (r: 0.240, P = 0.001), and 5-time chair stand test (r: 0.247; P<0.001). Conversely, VPT is negatively correlated with fasting insulin (r: -0.168, P = 0.019), HDL-c (r: -0.194, P = 0.006), and 6-m walk speed (r: -0.245; P<0.001) (**Table 3**).

**Table 3:** Correlation coefficients between VPT and various factors.

	VPT(V)	
	r	P
**Gender**	0.164	0.022
**Age**	0.363	<0.001
**Body weight**	0.181	0.011
**Drinker**	0.122	0.088
**Combined hypertension**	0.166	0.020
**HbA1c**	0.240	0.001
**FINS**	-0.168	0.019
**HDL-c**	-0.194	0.006
**Creatinine**	0.209	0.003
**5-time chair stand test**	0.247	<0.001
**6-m walk speed**	-0.245	<0.001

HbA1c, glycosylated hemoglobin; FINS, fasting insulin; HDL-c, serum high-density lipoprotein.

## Discussion

In our MR analysis, we investigated the relationship between diabetic neuropathy and parts of the relevant clinical traits of sarcopenia. The results suggest a partial causality between sarcopenia and diabetic neuropathy. Moreover, a cross-sectional study was carried out to further investigate the correlation between DPN and sarcopenia in T2DM patients, revealing that patients with DPN have a significantly higher prevalence of sarcopenia than those without sarcopenia. In addition, this study also suggested that sarcopenia indexes were independent risk factors for DPN, which is generally consistent with our MR analysis findings.

A previous cross-sectional study found that diabetic patients with DPN exhibited slower walking speeds and greater variability in step frequency compared to diabetic patients without DPN and healthy controls (16). Additionally, a cohort study from the UK Biobank showed that T2DM participants with faster walking speeds had a lower incidence of DPN (40). These findings are consistent with the results of our MR analysis and cross-sectional study. A single-center study from China found that DPN impairs hand function, primarily manifesting as reduced fingertip grip strength and decreased hand dexterity (41). While our reverse MR analysis supports these findings, our forward MR analysis did not show a significant association between hand grip strength and diabetic neuropathy. Another study reported that patients with more severe diabetic foot neuropathy had lower ALM index (42). This conclusion is contrary to our results, which might be due to factors such as insufficient sample size or the use of samples exclusively from a single European population. A previous MR study indicated that ALM did not show a significant causal relationship with T2DM, which is consistent with our findings (43).

Several previous studies have demonstrated a correlation between sarcopenia and diabetic neuropathy, yet the underlying mechanisms by which diabetic neuropathy leads to the development of sarcopenia remain unclear. Andreassen et al. demonstrated that DPN induces muscle atrophy and weakness through muscle denervation resulting from motor axon loss (44). Zhang et al. found that increased muscle mass can partially improve sensory and motor nerve conduction velocity (45). On the other hand, Han et al. observed that the low muscle mass seen in sarcopenic patients can lead to decreased glucose uptake and increased insulin resistance (46). Jarmusch et al., showed that some muscle-secreted cytokines, such as insulin-like growth factor 1, have a major nutritional impact on neurons. Lower levels of insulin-like growth factor-1 expression in sarcopenia have been linked to muscle denervation, as well as poor neuronal regeneration, potentially contributing to the onset of sarcopenia (47).

This study still had some disadvantages. In terms of MR analysis, firstly, past study showed that sarcopenia may be influenced by sex and age, but the GWAS database lacks sex and age stratification, which limits our ability to perform subgroup analyses, such as those regarding low hand-grip strength and ALM according to sex and age. Secondly, due to the lack of ASMI data in the GWAS database, we had to use another widely accepted measure, ALM, as a substitute for ASMI in calculating muscle mass. However, it may not be insufficiently precise due to deviations in other non-fat soft tissues such as lungs and kidneys. Thirdly, our MR analysis included only European populations, which may not be applicable enough to groups from other regions. Lastly, residual bias cannot be completely avoided, as it is a known limitation of the MR technique. Despite the use of pleiotropy tests and MR-PRESSO procedures to minimize confounding by pleiotropy, some bias may still remain. In terms of our cross-sectional study, our participants were selected from hospitalized patients at a single center, consisting only of patients over 60 years old with T2DM, which may lead to selection bias. Additionally, due to the lack of relevant diagnostic equipment, the research did not include Nerve conduction studies to diagnose DPN, which are considered the current gold standard. Using only VPT to explore the correlation between DPN and sarcopenia without including assessments related to autonomic neuropathy, might not be entirely convincing. Indeed, more samples from different locations are required to demonstrate the precise link between DPN and sarcopenia more thoroughly. Nevertheless, despite these limitations, it is still necessary for clinical doctors to screen for sarcopenia in patients with DPN. The easy-to-use VPT test not only helps diagnose DPN but also indicates the risk of abnormal body functions, which can help T2DM patients prevent falls, fractures, and even foot diseases.

In conclusion, this study provides solid evidence that clinical traits of sarcopenia, including VPT, walking speed, hand grip strength and AMSI, are possible predictors of diabetic neuropathy. Nevertheless, despite some limitations, it is still necessary for clinical doctors to screen for sarcopenia in patients with DPN. The easy-to-use VPT test not only helps diagnose DPN but also indicates the risk of abnormal muscle functions, which can help T2DM patients prevent falls, fractures, and even foot diseases.

## Data Availability

The datasets presented in this study can be found in online repositories. The names of the repository/repositories and accession number(s) can be found in the article/**Supplementary Material**.
